# On the Influence of Opium upon the Catamenial Functions

**Published:** 1844-02

**Authors:** 


*On the Influence of Opium upon the Catamenial Functions.*— In the same number of the New York Journal we find a report of several cases in which the catamenial discharge was suppressed by the use of opium. The facts are novel, and may lead to valuable results in practice:

'Case I.—E. L——d, a middle sized, plethoric young woman, *aet.* 24. In 1837, having procured an abortion by means of some powerful drug, she sent for a physician, who gave opiates in the course of his subsequent treatment. From that time until April, 1843, she resorted daily to laudanum, and gradually increased the dose to four ounces per diem. Her appearance last April was as follows: look, stupid; complexion, florid; person, fat; bowels, regular; and she ate very little food. Her catamenia, which had been regular until January, 1837, have never appeared since. I believe she is now in the Magdalen Asylum at Yorkville.

Case II.—E. M——l, *aet.* 23, of middle size and sanguine temperament. About three and a half years ago, during an attack of acute rheumatism, laudanum was freely administered to her. During her recovery, and until March, 1843, she continued the daily use of the tincture of opium; and from 1841 to 1843, she drank from four to six ounces per day. In March last, she had clear complexion, ruddy cheeks, good general health, bowels regular, and ate little food; but from the date of her rheumatism, until March, 1843, her catamenia did not appear, notwithstanding she had been perfectly 'regular' until that attack. She went into the country, in April, 1843, where she could not obtain any laudanum; but she applied to a physician, who prescribed an emmenagogue, which was followed, at first, by a slight, and subsequently, by several profuse, but regular, returns of the catamenia. She has recently (July 20th) returned to this city, and resumed a very moderate allowance of laudanum.

"Case III.—S. S——h, a short, fleshy, plethoric young woman, (colored), *aet.* 26. Until July, 1837, she had menstruated regularly; but, at that date, she commenced the daily use of laudanum to allay the pain of *onychia maligna* of the great toe of the left foot. She continued drinking from two to four ounces of tr. opii, per diem, until June, 1842. During these five years, although her general health remained unimpaired, her catamenia did not appear. At the last-named date, she was cured of the *onychia*, by an operation. The succeeding month, and every month since, the menses have punctually appeared, without the aid of any medicine.

"Case IV.—R——n, *aet.* 25, of middle height, robust, sanguine temperament; has a child four years old. About two years ago, while confined to her room by a fracture of the left tibia, she commenced using opium to relieve pain and restlessness. She has sometimes used twenty or thirty grains of opium per day, for two or three months steadily; at other times, she quitted the stimulant entirely, for the same length of time. While she continued using the opium daily, her catamenia did not appear; but, on forsaking the drug for a month or two, she becomes 'regular' without an emenagogue. Her general health is good, and she has not been constipated at any time.

"Case V.—E. R——, *aet.* 18, a young, unmarried female (colored), applied to me in May or June, 1841, for relief from an increase in the quantity and frequency of the catamenia. She was pale, thin, and of the nervo-sanguine temperament. Pulse frequent, but soft; head dizzy. She has a sister and brother; both are married and have progeny. There were no coagula in her uterine discharges, and except physical weakness, her general health was good. Having tried the ordinary treatment without success, I gave her a combination of acetate of lead, camphor, and *opium*, in minute, frequently repeated, doses. I commenced the use of this combination on the fourth day of the persistence of the discharge, and continued the remedy until the discharge ceased. She was greatly relieved by this practice. Shortly afterwards, on leaving the city, she procured a copy of the prescription; and, without my knowledge, continued using the pills (as her sister informed me lately) nearly a year. The catamenia gradually diminished, and, at the end of a year, entirely ceased. She was subseqnently (Sept. 1842) married. Her general health remains good; her person is thin; but menstruation has not recurred, nor has she yet become pregnaut. Since abstaining from the pills (Aug. 1842), she has repeatedly resorted to emmenagogues, prescribed by various physicians, but without success.

"*Remarks.*—The first four of the above females are courtezans. But their vocation cannot have been the cause of the arrest of their menses; for I can say, from a somewhat extensive experience in the Lock Hospital, at Glasgow, that this unfortunate class have no peculiar liability to suppressio mensium. E. M——l (case II) informed me that she is acquainted with several opium-eaters, in all of whom the menses have been arrested, as they believe, by opium.

"Without assuming from so few cases, that the daily use of opium will *always* arrest the catamenia, I submit them, in the hope that they may attract the attention of medical men to whatever facts may be within their reach. It will be interesting to ascertain, in relation to married women, who have borne children previously to a resort to opium-eating, whether this habit arrests their catamenia, and their capacity for conception; or whether it arrests the one and not the other. It may also be worth the inquiry, whether opium, in skilfully regulated doses, may not be used as a means to bear women safely through the critical disturbances which occur at the 'change of life.'

"One circumstance is common to all the above narrated cases, viz., the arrest of the catamenia was not followed by the vital disturbances which attend suppression from other causes; or, in general terms, opium, in daily doses, arrested uterine periodicity without disturbing other ordinary vital functions. Here we have an analogy with other well known effects of opium; for this drug, to a great degree, arrests hunger, which, in the present state of civilized society, is a *periodic habit*, for it is seldom allowed to become an actual *want.* In a therapeutical point of view, opium exerts an almost uniform influence as an arrester of periodicity. As a narcotic, its influence is not uniform, for it does not always produce sleep; and I am inclined to the belief, that when sleep does follow its exhibition, it is rather a consequence of the absence of (an arrested paroxysm) than of a direct narcotic influence."

